# Synthesis and electroluminescence property of new hexaphenylbenzene derivatives including amine group for blue emitters

**DOI:** 10.1186/1556-276X-8-421

**Published:** 2013-10-17

**Authors:** Hwangyu Shin, Yun-Fan Wang, Jong-Hyung Kim, Jaehyun Lee, Kwang-Yol Kay, Jongwook Park

**Affiliations:** 1 Department of Chemistry, The Catholic University of Korea, Bucheon 420-743, South Korea; 2 Department of Molecular Science and Technology, Ajou University, Suwon 443-749, South Korea

**Keywords:** Organic light-emitting diode, Blue-emitting materials, Hole transporting

## Abstract

Three new blue-emitting compounds of 5P-VA, 5P-VTPA, and 5P-DVTPA for organic light-emitting diode (OLED) based on hexaphenylbenzene moiety were demonstrated. Physical properties by the change of the substitution groups of the synthesized materials were systematically examined. Photoluminescence spectrum of the synthesized materials showed maximum emitting wavelengths of about 400 to 447 nm in solution state and 451 to 461 nm in film state, indicating deep blue emission color. OLED devices were fabricated by the synthesized compounds using vacuum deposit process as an emitting layer. The device structure was ITO/2-TNATA 60 nm/ NPB 15 nm/ EML 35 nm/ TPBi 20 nm/ LiF 1 nm/ Al 200 nm. External quantum efficiencies and CIE values of 5P-VA, 5P-VTPA, and 5P-DVTPA were 1.89%, 3.59%, 3.34%, and (0.154, 0.196), (0.150, 0.076), (0.148, 0.120), respectively. 5P-VTPA and 5P-DVTPA exhibited superior highly blue quality and thermal property such as high *T*_d_ of 448°C and 449°C.

## Background

Intensive studies have been conducted on organic light-emitting diodes (OLEDs) as they have a great potential to be applied to large full-color displays and mobile displays [1-3]. Most of the conjugated organic molecules have been reported as red, green, and blue electroluminescence (EL) [4]. It is required for those red, green, and blue emitters to show high EL efficiency, good thermal properties, long lifetime, and pure color coordinates (1931 Commission Internationale de l’Eclairage (CIE)) in order to be applied to large full-color displays. A red light-fluorescence emitter with CIE coordinates of (0.66, 0.34) and a long lifetime of more than 600,000 h at 24 cd/A has recently been developed. A green light-fluorescence emitter with CIE coordinates of (0.34, 0.62) and a lifetime of 400,000 h at 78 cd/A has also been achieved [5]. However, the best official results for a blue-light emitter are a short lifetime of only 10,000 h at 9.0 cd/A and CIE coordinates of (0.14, 0.12) with fluorescence materials [6]. Thus, the development of a blue emitter with high color purity, high efficiency, and a long lifetime is an extremely challenging research topic. Most existing studies of blue emitters use molecules with excellent fluorescence characteristics such as anthracene [7,8] and pyrene [9,10] as core or side moieties. Many studies have investigated the use of anthracene and pyrene as blue core moiety since they have high photoluminescence (PL) and EL efficiencies. However, these molecules can easily form excimers through packing because anthracene and pyrene have flat molecular structure that reduce EL efficiency and degrade color purity [11].

**Figure 1:**
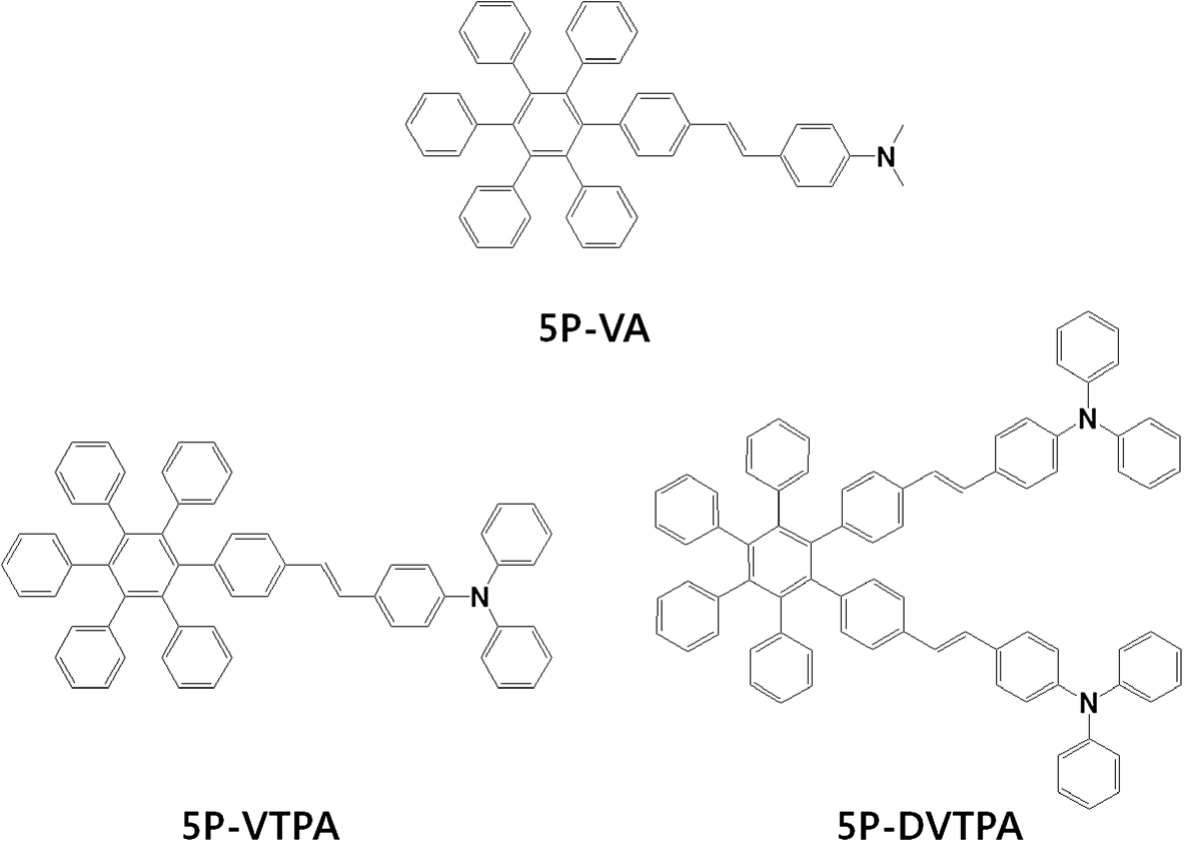
Chemical structures of 5P-VA, 5P-VTPA, and 5P-DVTPA.

In this work, new blue-emitting compounds based on hexaphenylbenzene group are designed and synthesized as shown in Figure 1. Aromatic amine moiety as a side group was introduced into main core structure in order to prevent intermolecular interaction and improve hole mobility [12]. Also, the change of emission wavelength as well as device efficiency was evaluated according to the different side group.

## Methods

Reagents and solvents were purchased as reagent grade and used without further purification. All reactions were performed using dry glassware under nitrogen atmosphere. Analytical TLC was carried out on Merck 60 F254 silica gel plate, and column chromatography was performed on Merck 60 silica gel (230 to 400 mesh) (Merck & Co., Inc., Whitehouse Station, NJ, USA). Melting points were determined on an Electrothemal IA 9000 series melting point apparatus (Bibby Scientific Limited, Stone, Staffordshire, UK) and are uncorrected. Nuclear magnetic resonance (NMR) spectra were recorded on a Varian Mercury-400 (400 MHz) spectrometer (Varian Medical Systems Inc., Palo Alto, CA) with TMS peak as reference. The optical absorption spectra were obtained by HP 8453 UV–vis-NIR spectrometer (HP Company, Palo Alto, CA, USA). Thermal properties of the compounds were measured by thermogravimetric analysis (TGA) and differential scanning calorimeter (DSC) using a SDT2960 and DSC2910 (TA Instruments, New Castle, DE, USA). Voyager-DE-STR, elemental analysis was performed with a PerkinElmer 2400 analyzer (PerkinElmer, Waltham, MA, USA). PerkinElmer luminescence spectrometer LS50 (Xenon flash tube) was used for PL spectroscopy. Surface analyzer AC-2 (RIKEN KEIKI, Itabashi-ku, Tokyo, Japan) was used for work function measurement. EL devices were fabricated as the following structure: ITO/ 2-TNATA 60 nm/ NPB 15 nm/ EML 35 nm/ TPBi 20 nm/ LiF 1 nm/ Al 200 nm, where 4,4′,4″-tris(*N*-(2-naphthyl)-*N*-phenyl-amino)-triphenylamine (2-TNATA) was used as a hole injection layer, *N*,*N*’-bis(naphthalene-1-ly)-*N*,*N*’-bis(phenyl)benzidine (NPB) as a hole transporting layer, the synthesized materials as emitting layer (EML), 1,3,5-tri(1-phenyl-1H-benzo[d]imidazol-2-yl)phenyl (TPBi) as an electron transporting layer and hole blocking layer, lithium fluoride (LiF) as an electron injection layer, ITO as anode, and Al as cathode. The organic layer was vacuum deposited by thermal evaporation at a vacuum base pressure of 10^-6^ Torr and the rate of deposition being 1 Å/S to give an emitting area of 4 mm^2^, and the Al layer was continuously deposited under the same vacuum condition. The current–voltage-luminance (I-V-L) characteristics of the fabricated EL devices were obtained using a Keithley 2400 electrometer (Keithley Instruments Inc, Solon, OH, USA), and light intensity was obtained using Minolta CS 1000A (Minolta Co., Ltd., Chuo-ku, Osaka, Japan).

### Synthesis of hexaphenylbenzene-based compounds 1, 2, and 3

**Figure 2:**
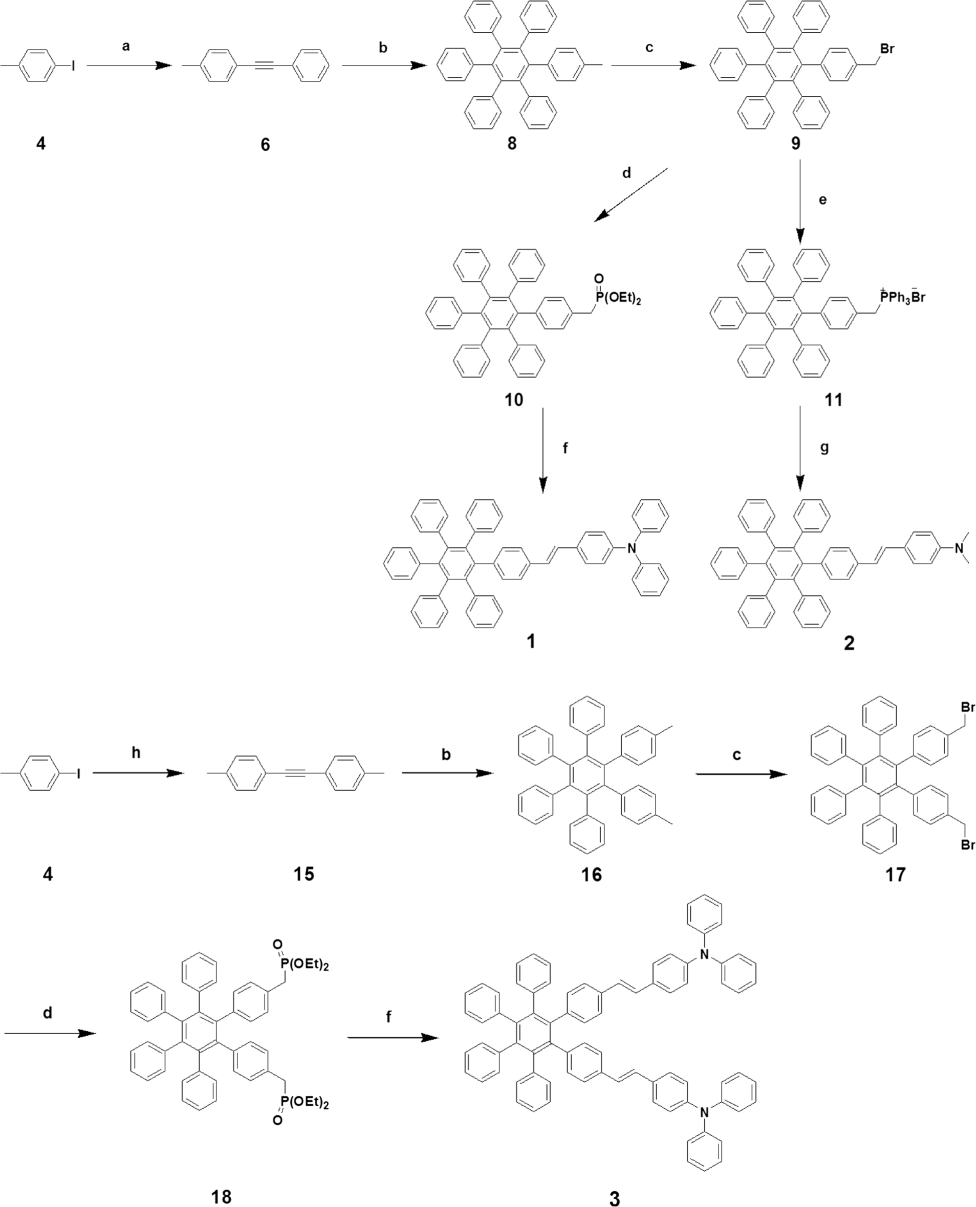
**Synthesis of compounds 1, 2, and 3. (a)** Phenylacetylene (**5**), Pd(PPh_3_)_2_Cl_2_, CuI, (Et)_3_ N, 50°C, 1 h, 92.5%. **(b)** Tetraphenylcyclopentadienone (**7**), diphenyl ether, reflux, 48 h, 78.6% for **8**, 72.6% for **16**. **(c)***N*-Bromosuccinimide (NBS), 2,2′-azobis(2-methylpropionitrile) (AIBN), CCl_4_, reflux, 4 h, 75.8% for **9**, 78.0% for **17**. **(d)** P(OEt)_3_, reflux, 24 h, 74.0% for **10**, 82.0% for **18**. **(e)** PPh_3_, DMF, reflux, 24 h, 64.0%. **(f)** 4-(Diphenylamino)benzaldehyde (**12**), NaH, THF, rt, 36 h, 40.0%. **(g)** 4-(Dimethylamino)benzaldehyde (**13**), NaOt-Bu, MeOH, reflux, 24 h, 36.0%. **(h)** 1-ethynyl-4-methylbenzene (**14**), Pd(PPh_3_)Cl_2_, CuI, Et_3_N, 50°C, 1 h, 92.3%.

The most straight-forward preparation of compounds **1**, **2**, and **3** can be envisaged to proceed through a reaction sequence of the following steps, as depicted in Figure 2. Every step of the reaction sequence proceeded smoothly and efficiently to give a good or moderate yield of the product (see the experimental section for the synthetic details). Commercially available 4-iodotoluene (**4**) was reacted with phenylacetylene (**5**) through Sonogashira coupling [13-15] to give **6** in 92.5% yield, and then, the subsequent cyclization with tetraphenylcyclopentadienone through Diels-Alder reaction [16] was carried out to give compound **8** in 78.6%. Compound **8** was brominated and phosphonated to produce compound **10** in 74.0%. Typical Wittig-type reactions of aldehydes **12** and **13** with **10** and **11** gave **1** and **2** in 40.0% and 36.0% yield, respectively. It is noticeable that phosphonation yield of bromocompound **9** was 10% higher than the ylid formation of **9**, and the subsequent Wittig-Horner-Emmons reaction of aldehyde **12** with the phosphonate **10** gave a better yield than the reaction of aldehyde **12** with the ylid **11** (Wittig reaction). On the other hand, the reaction of aldehyde **13** with ylid **11** produced a better yield than the reaction of **13** with **10** for the synthesis of **2**. Even although we have not optimized the above both reactions, we had to choose the Wittig-Horner-Emmons-type reaction for **1** and Wittig reaction for **2** after several trials. Accordingly, analogously prepared hexaphenylbenzene-based diphosphonate **18** reacted with aldehyde **12** to produce **3** in 32.0% yield.

Compounds **1**, **2**, and **3** and their precursor compounds are very soluble in aromatic solvents (i.e., toluene, o-dichlorobenzene, and benzonitrile) and other common organic solvents (i.e., acetone, CH_2_Cl_2_, CHCl_3_, and THF). The structure and purity of the newly synthesized compounds were confirmed mainly by ^1^H NMR and elemental analysis. ^1^H NMR spectra of **1**, **2**, and **3** are consistent with the proposed structures, showing the expected features with the correct integration ratios, respectively. The matrix-assisted laser desorption/ionization-time-of-flight (MALDI-TOF) mass spectra provided a direct evidence for the structures of **1**, **2**, and **3,** showing a singly charged molecular ion peaks at *m*/*z* = 803.38 for **1**, *m*/*z* = 679.35 for **2**, and *m*/*z* = 1,073.24 for **3**, respectively.

### 4-Methylphenylphenylacetylene (6)

To a mixture of 4-iodotoluene (**4**) (1.0 g, 4.6 mmol), dichlorobis(triphenylphosphine)palladium(II) (32 mg, 0.046 mmol), and copper iodide (9 mg, 0.046 mmol) in triethylamine (60 ml), phenylacetylene (**5**) (0.36 ml, 5.52 mmol) was added and stirred at 50°C for 1 h. The solvent was evaporated under reduced pressure, and the residue was chromatographed on silica gel with hexane to give **6** (0.81 g, 92.5%) in a white solid. M.p. 67°C to 69°C. ^1^H NMR (400 MHz, CDCl_3_): *δ* = 2.38 (s, 3H), 7.16(d, *J* = 8.8 Hz, 2H), 7.35 (m, 3H), 7.45 (d, *J* = 8.8 Hz, 2H), 7.55 (m, 2H). Anal. Calcd for C_15_H_12_: C, 93.70%; H, 6.29%. Found: C, 93.59%; H, 6.41%.

### Pentaphenylphenyl-4-methylbenzene (8)

Compound **6** (1.11 g, 5.78 mmol) and tetraphenylcyclopentadienone (**7)** (2.67 g, 7.0 mmol) were dissolved in diphenyl ether (30 ml), and the mixture was refluxed for 48 h. The solvent was evaporated under reduced pressure, and the residue was recrystallized from ethanol to afford **8** (2.54 g, 78.6%) in a yellow-gray solid. M.p. 370°C to 372°C. ^1^H NMR (400 MHz, CDCl_3_): *δ* = 2.00 (s, 3H), 6.56 (d, *J* = 8.8 Hz, 2H), 6.62 (d, *J* = 8.8 Hz, 2H), 6.76 (m, 25H). Anal. Calcd for C_43_H_32_: C, 94.12%; H, 5.88%. Found: C, 93.96%; H, 6.04%.

### Pentaphenylphenyl-4-bromomethylbenzene (9)

A mixture of compound **8** (0.83 g, 1.5 mmol), *N*-Bromosuccinimide (NBS, 0.32 g, 1.8 mmol), and 2,2′-azobis(2-methylpropionitrile (AIBN, 0.124 g, 0.76 mmol) in CCl_4_ (125 ml) was refluxed for 4 h. After cooling to the room temperature, the solvent was evaporated under reduced pressure, and then, the residue was chromatographed on silica gel with dichloromethane/hexane (1:2) to give a white solid in a yield of 0.72 g (75.8%). M.p. 271°C. ^1^H NMR (400 MHz, CDCl_3_): *δ* = 4.22 (s, 2H), 6.70 (m, 29H). Anal. Calcd for C_43_H_31_Br: C, 82.29%; H, 4.98%. Found: C, 82.12%; H, 5.13%.

### Pentaphenylphenyl-4-diethylphosphomethylbenzene (10)

The mixture of **9** (0.20 g, 0.31 mmol) and triethylphosphate (10 ml) was refluxed for 24 h. The solvent was evaporated under reduced pressure, and the residue was recrystallized from hexane. The precipitate was filtered and dried in vacuum oven to give **10** (0.16 g, 74.0%) in a white solid. M.p. 239°C. ^1^H NMR (400 MHz, CDCl_3_): *δ* =1.10 (t, *J* = 6.8 Hz, 6H), 2.90 (s, 2H), 3.77 (q, *J* = 6.8 Hz, 4H), 6.70 (m, 29H). Anal. Calcd for C_47_H_41_PO_3_: C, 82.43%; H, 6.04%. Found: C, 82.17%; H, 6.13%.

### Pentaphenyl(4-methylphenyl)benzene-triphenylphosphonium bromide (11)

A mixture of **9** (5.0 g, 7.8 mmol) and triphenylphosphine (2.47 g, 9.4 mmol) in dimethylformamide (DMF; 150 ml) was refluxed for 24 h. After cooling to room temperature, the mixture was quenched with ether. The precipitates were filtered and recrystallized from dichloromethane/hexane (1:1) to give **11** (4.5 g, 64.0%) in a white solid. ^1^H NMR (400 MHz, CDCl_3_): *δ* = 3.00 (s, 2H), 6.45 to 6.90 (m, 29H), 7.32 to 7.80 (m, 15H). Anal. Calcd for C_61_H_46_PBr: C, 82.33%; H, 5.21%. Found: C, 82.09%; H, 5.34%.

### 4-{4-(Diphenylaminophenyl)-ethenyl}phenylpentaphenylbenzene (1)[5P-VTPA]

A mixture of compound **10 (**0.3 g, 0.44 mmol), 4-(diphenylamino)benzaldehyde (**12**) (0.10 g, 0.37 mmol), and sodium hydride (0.3 g, 13 mmol) in anhydrous THF (100 ml) was stirred at room temperature for 72 h. The reaction mixture was quenched with water (300 ml) and then extracted with dichloromethane (3 × 100 ml). After the evaporation of organic extracts, the residue was chromatographed on silica gel with dichloromethane/hexane (1:2) to give **1** (0.3 g, 40.0%) in a yellow solid. M.p. 294°C. ^1^H NMR (400 MHz, CDCl_3_): *δ* = 6.70 to 6.90 (m, 25H), 6.92 to 7.05 (m, 6H), 7.05 to 7.09 (m, 4H), 7.14 to 7.24 (m, 10H). ^13^C NMR (CDCl_3_): *δ* = 122.60, 122.73, 123.20, 123.24, 123.41, 124.03, 124.25, 125.20, 126.73, 126.87, 127.31, 127.41, 127.71, 127.85, 127.93, 129.32, 129.61, 129.72, 131.19, 131.55, 131.78, 134.07, 134.30, 135.72, 136.51, 140.28, 141.06. MS (MALDI-TOF): *m*/*z* for C_62_H_45_N Calcd 803.98. Found 803.38 (M^+^). Anal. Calcd for C_62_H_45_N: C, 92.62%; H, 5.64%; N, 1.74%. Found: C, 92.41%; H, 5.76%; N, 1.83%.

### 4′-(4-Dimethylamino)styrylhexaphenylbenzene (2)[5P-VA]

A mixture of **11** (1.4 g, 1.57 mmol), 4-(dimethylamino)benzaldehyde (**13**) (0.4 g, 2.7 mmol), and sodium tert-butoxide (1.1 g, 11.2 mmol) in methanol (80 ml) was refluxed for 24 h. After the evaporation of the solvent under reduced pressure, the residue was chromatographed on silica gel with dichloromethane/hexane (1:1) to give **2** (0.43 g, 36.0%) in a white solid. M.p 298°C. ^1^H NMR (400 MHz, CDCl_3_): *δ* = 2.80 (s, 6H), 6.50 to 6.74 (m, 4H), 6.74 to 6.80 (m, 25H), 6.86 (m, 4H), 7.18 (d, *J* = 8.8 Hz, 2H). ^13^C NMR (CDCl_3_): *δ* = 40.32, 112.81, 124,72, 124.83, 125.21, 125.34, 125.87, 126.04, 126.74, 126.78, 126.91, 127.07, 127.12, 127.40, 127.65, 127.74, 127.92, 129.34, 130.04, 131.84, 131.92, 135.30, 139.53, 140.48, 140.92, 140.97, 149.78. MS (MALDI-TOF): *m*/*z* for C_52_H_41_N Calcd 679.36. Found 679.35 (M^+^). Anal. Calcd for C_52_H_41_N: C, 91.86%; H, 6.08%; N, 2.06%. Found: C, 91.62%; H, 6.19%; N, 2.19%.

### Bis(4-methylphenyl)acetylene (15)

To a mixture of 4-iodotoluene (**4**) (2.24 g, 10.3 mmol), dichlorobis(triphenylphosphine)palladium (II) (0.11 g, 0.09 mmol), and copper iodide (16 mg, 0.086 mmol) in triethylamine (60 ml), 4-acetyltoluene (**14**) (1.0 g, 8.60 mmol) was added and stirred at 50°C for 1 h. The solvent was evaporated under reduced pressure, and the residue was chromatographed on silica gel with hexane to give **15** (1.63 g, 92.3%) in a white solid. M.p. 73°C. ^1^H NMR (400 MHz, CDCl_3_): *δ* = 2.30 (s, 6H), 7.00 (d, *J* = 8.4 Hz, 4H), 7.30 (d, *J* = 8.4 Hz, 4H). Anal. Calcd for C_16_H_14_: C, 93.16; H, 6.84%. Found: C, 92.99%; H, 7.01%.

### 1,2-Di(4-methylphenyl)-3,4,5,6-tetraphenylbenzene (16)

Compound **15** (1.64 g, 8.00 mmol) and tetraphenylcyclopentadienone (**7**) (3.67 g, 9.50 mmol) were dissolved in diphenyl ether (20 ml), and the mixture was refluxed for 48 h. After cooling to room temperature, the mixture was poured into ethanol (800 ml) and stirred for 4 h. The precipitates thus obtained were dried to give **16** (3.24 g, 72.6%) in a gray solid. M.p. 313°C. ^1^H NMR (400 MHz, CDCl_3_): *δ* = 2.08 (s, 3H), 2.17 (s, 3H), 6.64 (d, *J* = 8.4 Hz, 4H), 6.68 (d, *J* = 8.4 Hz, 4H), 6.76 to 6.84 (m, 20H). Anal. Calcd for C_44_H_34_: C, 93.91%;H, 6.09%. Found: C, 93.77%; H, 6.23%.

### 1,2-Di(4-bromomethylphenyl)-3,4,5,6-tetraphenylbenzene (17)

A mixture of compound **16** (3.25 g, 5.80 mmol), NBS (2.48 g, 13.9 mmol), and AIBN (0.95 g, 5.80 mmol) in CCl_4_ (125 ml) was refluxed for 8 h. After cooling to room temperature, the solvent was evaporated under reduced pressure, and then the residue was chromatographed on silica gel with dichloromethane/hexane (1:2) to give a white solid in a yield of 3.26 g (78.0%). M.p. 257°C. ^1^H NMR (400 MHz, CDCl_3_): *δ* = 4.20 (s, 4H), 6.60 to 6.80 (m, 28H). Anal. Calcd for C_44_H_32_Br_2_: C, 73.34%; H, 4.45%. Found: C, 73.01%; H, 4.53%.

### 1,2-Di(4-diethylphosphomethylphenyl)-3,4,5,6-tetraphenylbenzene (18)

The mixture of **17** (3.26 g, 4.50 mmol) and triethylphosphate (10 ml) was refluxed for 24 h. After cooling to room temperature, the mixture was poured into hexane (200 ml). The precipitates were filtered and chromatographed on silica gel with dichloromethane/methanol (40:1) to give **18** (3.10 g, 82.0%) in a white solid. M.p. 233°C. ^1^H NMR (400 MHz, CDCl_3_): *δ* =1.20 (t, *J* = 6.8 Hz, 12H), 2.85 (s, 4H), 3.75 (q, *J* = 6.8 Hz, 8H), 6.64 to 6.80 (m, 28H). Anal. Calcd for C_52_H_52_P_2_O_6_: C, 74.80%; H, 6.28%. Found: C, 74.59%; H, 6.13%.

### 1,2-{Bis(4-diphenylamino)styryl}-3,4,5,6-tetraphenylbenzene (3)[5P-DVTPA]

To the mixture of **18** (2.88 g, 3.45 mmol) and compound **12** (2.26 g, 8.29 mmol) in THF(100 ml), NaH (0.3 g, 13 mmol) was added. The reaction mixture was then stirred at room temperature for 72 h. The mixture was quenched with water (300 ml) and then extracted with dichloromethane (400 ml). The organic layer was separated, and the solvent was evaporated under reduced pressure. The residue was chromatographed on silica gel with dichloromethane/hexane (1:2) to give **3** (1.2 g, 32.4%) in a yellow solid. M.p. 307°C. ^1^H NMR (400 MHz, CDCl_3_): *δ* = 6.70 to 6.86 (m, 28H), 6.90 to 7.10 (m, 18H), 7.20 to 7.32 (m, 14H). ^13^C NMR (CDCl_3_): *δ* = 122.61, 122.73, 123.23, 123.28, 124.89, 124.93, 125.14, 125.20, 126.93, 126.95, 127.30, 127.42, 127.73, 127.81, 127.88, 127.92, 127.95, 129.30, 129.63, 129.72, 131.57, 131.62, 131.74, 131.83, 134.11, 134.33, 140.34, 141.02,141.08. MS (MALDI-TOF): *m*/*z* for C_82_H_60_N_2_ Calcd 1,073.32. Found 1073.24 (M^+^). Anal. Calcd for C_82_H_60_N_2_: C, 91.75%; H, 5.63%; N, 2.61%. Found: C, 91.62%; H, 5.72%; N, 2.66%.

## Results and discussion

**Figure 3:**
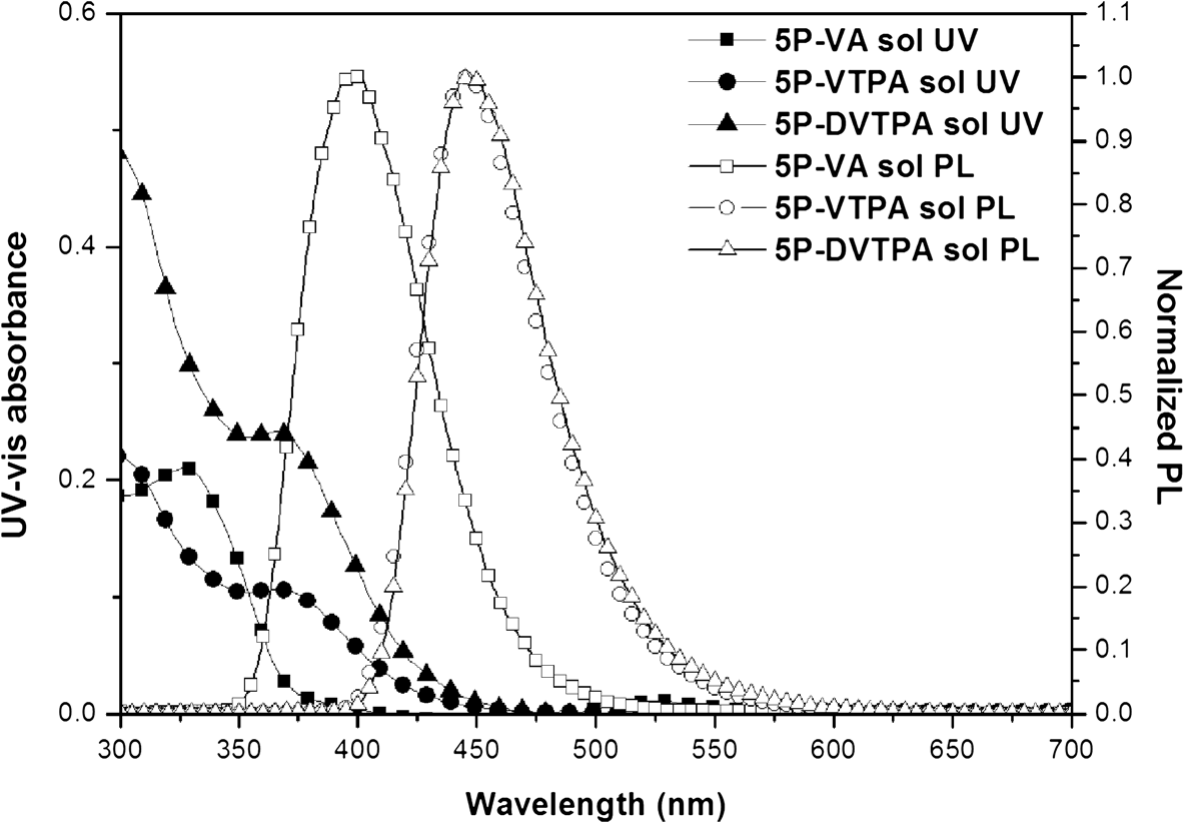
**UV–vis absorption spectra of 5P-VA (square, □), 5P-VTPA (circle, ○), 5P-DVTPA (triangle, △) in CHCl**_**3 **_**solution (1 × 10**^**-5 **^**M).**

**Figure 4:**
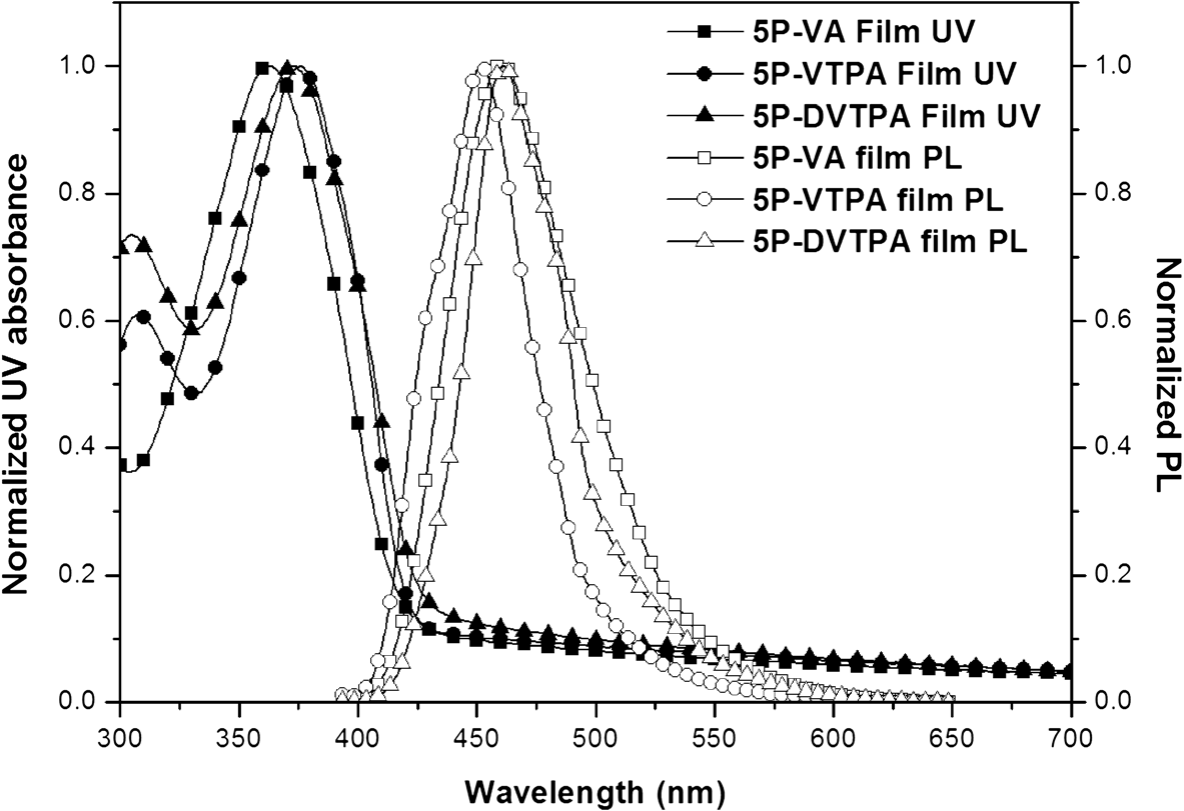
**UV–vis absorption spectra of 5P-VA (square, □), 5P-VTPA (circle, ○), 5P-DVTPA (triangle, △) in film state.** Film thickness is 50 nm.

The optical properties of synthesized compounds were summarized in Table 1 and Figures 3 and 4. Figure 3 shows ultraviolet–visible (UV–vis) absorption and PL spectrum data in solution state. Figure 4 exhibits spectrum data in film state. In the solution case, the solvent was used with chloroform (1 × 10^-5^ M), and 50 nm in thickness was chosen for evaporated film on glass. In Figure 3 and Table 1, 5P-VA had the longest maximum absorption and PL values of 327 and 400 nm, and 5P-VTPA and 5P-DVTPA had the longest maximum absorption values of 367 and 364 nm, and PL maximum (PL_max_) values of 446 and 447 nm, respectively.

**Table 1:** Optical properties of synthesized materials

**Compound**	**Solution**^**a**^	**Film**^**b**^	**Solution**^**a**^	**Film**^**b**^	***T***_**g**_	***T***_**m**_	***T***_**d**_
	**UV max(nm)**	**UV max(nm)**	**PL max(nm)**	**PL max(nm)**	**(°C)**	**(°C)**	**(°C)**
5P-VA	276, 327	363	400	460	100	312	388
5P-VTPA	301, 367	307, 376	446	451	108	309	448
5P-DVTPA	289, 364	305, 373	447	461	110	308	449

^a^10^-5^ M in chloroform, ^b^film thickness of 50 nm.

As shown in Figure 4 and Table 1, 5P-VA film showed maximum absorption value at 363 nm as well as PL_max_ value at 460 nm. In the 5P-VTPA and 5P-DVTPA cases, two compounds showed similar absorption maximum values at 376 and 373 nm, but PL_max_ values were slightly different as 451 and 461 nm.

In the case of 5P-VA, PL_max_ value was large red-shifted from 400 to 460 nm according to film state. It means that intermolecular distance in film state was closed, and intermolecular π-π* interaction was increased because of no bulky side group. However, 5P-VTPA and 5P-DVTPA having bulky side group of aromatic amine moiety had slightly red-shifted with 5 to 15 nm in film state.

5P-VTPA including diphenyl amine group in solution state showed large red shift of 46 nm in emission wavelength compared to 5P-VA having only alkyl amine and dimethyl amine (see Table 1).

DSC and TGA analyses to determine the thermal properties of the synthesized molecules were carried out (see Table 1). High *T*_g_ and *T*_d_ values indicate that the morphology of the material will not easily be changed by the high temperatures generated during the operation of OLED devices and are closely correlated with long OLED device life-times [17,18]. Two compounds showed high *T*_g_ of 108°C and 110°C and high *T*_d_ of 448°C and 449°C. Comparing on *T*_m_ and *T*_d_ of three compounds, two compounds having prevented molecular packing had the slightly decreased *T*_m_ and the increased *T*_g_ and *T*_d_. The increased *T*_g_ and *T*_d_ can be interpreted by the increased molecular weight.

Energy levels of three synthesized compounds such as HOMO, LUMO, and bandgap were estimated by ultraviolet photon spectroscopy of AC-2 and optical absorption spectroscopy (see Table 2). 5P-VA had HOMO and bandgap values of -5.50 and 2.99 eV, respectively. 5P-VTPA and 5P-DVTPA showed HOMO values of -5.65 and -5.60 eV and bandgap values of 2.95 and 2.89 eV, respectively. Bandgap was decreased and emission wavelength was red-shifted according to the change from alkyl amine side group to aromatic amine side group.

**Table 2:** **EL performance of multilayered devices with the synthesized compounds at 10 mA/cm**^**2**^

**Compound**	**Volt (V)**	**Current efficiency (cd/A)**	**Power efficiency (lm/W)**	**EQE (%)**	**CIE ( *x *, *y*)**	**EL maximum**	**HOMO (eV)**	**LUMO (eV)**	**Bandgap**
5P-VA	9.51	1.91	0.76	1.89	0.154, 0196	466	-5.50	-2.52	2.99
5P-VTPA	7.31	1.30	0.63	3.59	0.150, 0.076	451	-5.65	-2.70	2.95
5P-DVTPA	7.87	2.10	0.93	3.34	0.148, 0.120	457	-5.60	-2.71	2.89

Device: ITO/ 2-TNATA 60 nm/ NPB 15 nm/ EML 35 nm/ TPBi 20 nm/ LiF 1 nm/ Al 200 nm.

OLED devices of the three compounds as an EML were fabricated as ITO/2-TNATA 60 nm/NPB 15 nm/EML 35 nm/TPBi 20 nm/LiF 1 nm/Al 200 nm. All organic films were prepared by evaporation under high vacuum of 10^-6^ Torr.

**Figure 5:**
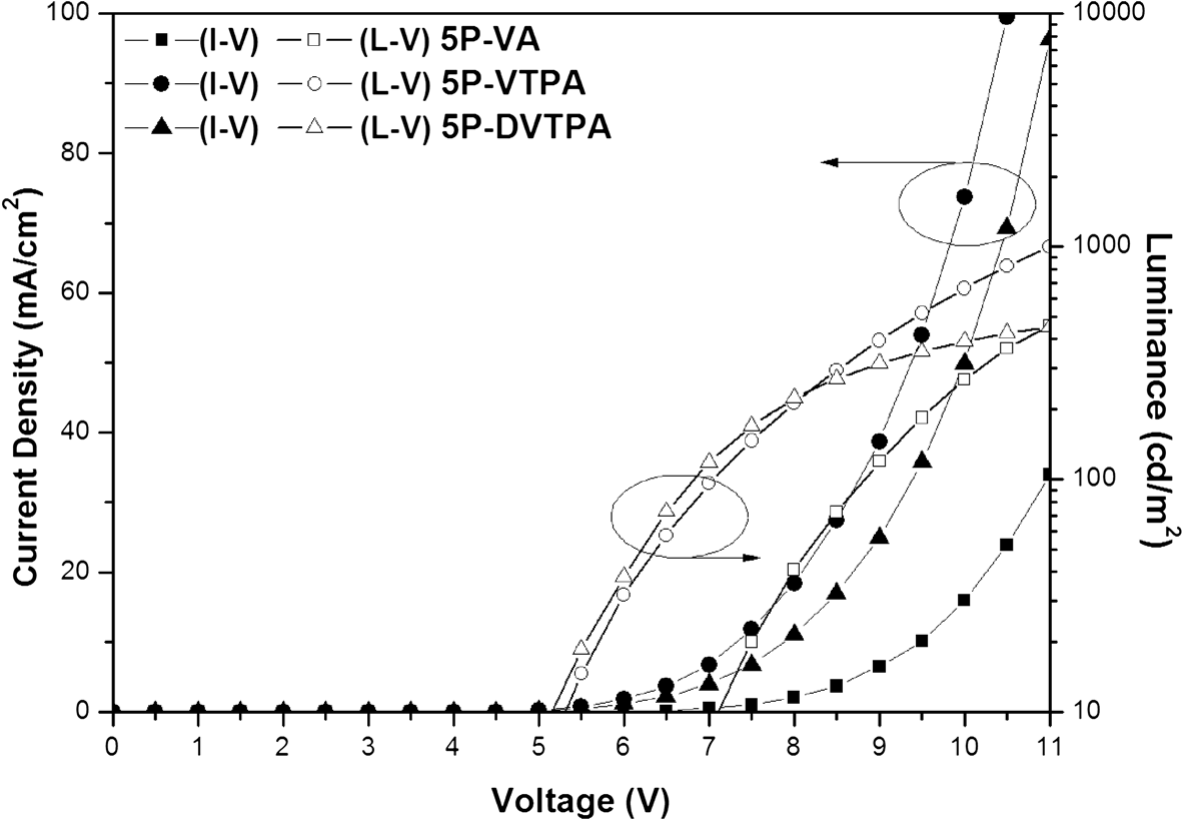
I-V-L graphs of 5P-VA, 5P-VTPA, and 5P-DVTPA OLED devices (device: ITO/ 2-TNATA 60 nm/ NPB 15 nm/ EML 35 nm/ TPBi 20 nm/ LiF 1 nm/ Al 200 nm).

**Figure 6:**
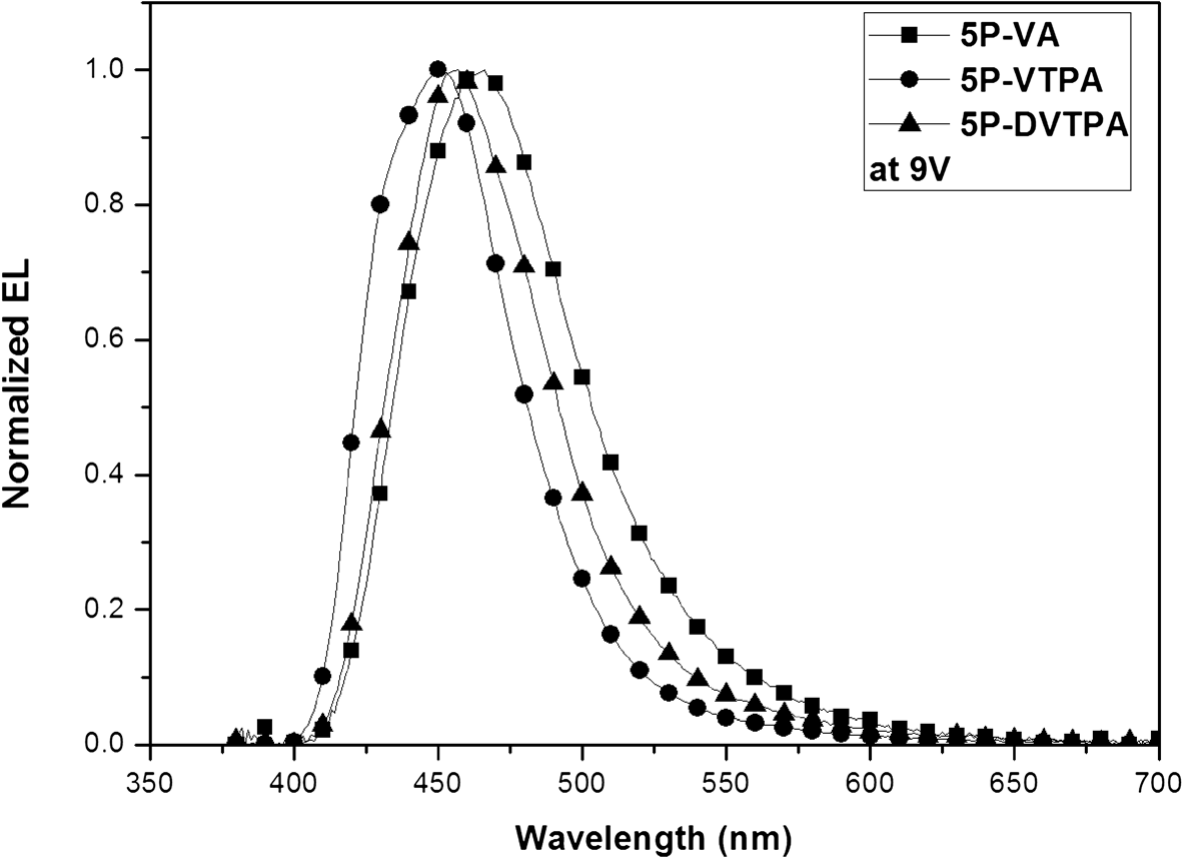
EL spectra of 5P-VA, 5P-VTPA, and 5P-DVTPA devices (device: ITO/ 2-TNATA 60 nm/ NPB 15 nm/ EML 35 nm/ TPBi 20 nm/ LiF 1 nm/ Al 200 nm).

Figure 5 shows I-V-L characteristics of the three devices. It exhibits the current density and luminance according to the applied voltage. I-V-L curves of the three compounds showed typical diode characteristics, but 5P-VTPA and 5P-DVTPA devices had the relatively smaller operating voltage compared to that of 5P-VA. The related efficiency data were also summarized in Table 2. Comparing external quantum efficiency (EQE) of the three devices, two compounds of 5P-VTPA and 5P-DVTPA had relatively 1.7 to 1.9 times higher efficiency than 5P-VA. Two devices of 5P-VTPA and 5P-DVTPA had low operating voltage such as 7.31 and 7.87 V although 5P-VA had lower energy barrier of HOMO level between NPB and EML because of small value of -5.50 eV. Low operating voltage might be explained by faster mobility of 5P-VTPA and 5P-DVTPA compared to 5P-VA, and it caused the increased efficiency. EL maximum values were shifted to deep blue, and CIE values showed excellent pure blue color *y* values of 0.076 and 0.120. Thus, aromatic amine side group prevented the packing of molecular structure, and it caused the improved blue color and EQE value. TV application is asking less than 0.08 *y* value for cold white OLED device, but it is extremely difficult to achieve that value. The normalized EL spectra of the three compound devices were shown in Figure 6.

## Conclusion

We demonstrated new blue fluorescence compounds based on hexaphenyl benzene derivatives. Those chemical structures can be varied by side groups of aliphatic and aromatic amine moiety. Three model compounds were designed and synthesized. Those were applied to OLED device as an EML, and the related properties were evaluated. Aromatic amine side groups can improve EL property such as color purity and operating voltage as well as EQE. 5P-VTPA, and 5P-DVTPA showed excellent CIE values of (0.150, 0.076), (0.148, 0.120) as a deep blue color. Especially, CIE value of 5P-VTPA can be applied to OLED TV application because of highly pure blue color. Also, 5P-VTPA and 5P-DVTPA exhibited superior thermal property such as high *T*_d_ of 448°C and 449°C.

## Competing interests

The authors declare that they have no competing interests.

## Authors’ contributions

HS carried out the synthesis and device characterization of the synthesized compounds. Y-FW carried out the synthesis of the synthesized compounds. J-HK synthesized one of the final compounds. JL carried out thermal property experiment of films. K-YK raised the idea of final chemical structures. JP suggested characterization methods and evaluation approach ways of the synthesized compounds. All authors read and approved the final manuscript.

## Authors’ information

HS is a Ph.D. course student for Organic Material Chemistry. Y-FW was a master course student for Organic Material Chemistry. J-HK was a Ph.D. course student for Organic Material Chemistry. JL is a Ph.D. course student for Organic Material Chemistry. K-YK is an emeritus professor of Organic Material Chemistry. JP is a full professor of Organic Material Chemistry and a director of the Display Research Center of The Catholic University of Korea.
